# Health information systems integrating patient-reported outcomes and experience: a scoping review

**DOI:** 10.1186/s41687-026-01146-6

**Published:** 2026-07-18

**Authors:** Pierre-Henri Roux-Levy, Marie-Eve Poitras, Wilfried Supper, Attisso Eugène, Marie-Ève Perron, Frédéric Bergeron, Michèle Dugas, Denis Roy, Vanessa Tremblay-Vaillancourt, Marie-Pierre Gagnon, Maxime Sasseville

**Affiliations:** 1 Department of Family Medicine and Emergency Medicine, University of Sherbrooke, Chaire de recherche sur les pratiques professionnelles optimales en soins primaires, Sherbrooke, Canada; 2 Faculty of Nursing Sciences, Université Laval, Quebec City, QC Canada; 3 Institut national de santé publique du Québec (INSPQ), Quebec City, QC Canada; 4 Université Laval, Quebec City, QC Canada; 5 Vitam Research Center on Sustainable Health, Quebec City, QC Canada; 6 Commissaire à la santé et au bien-être, Montréal, Qc Canada; 7 Chaire de recherche CRMUS sur les pratiques optimales professionnelles en soins primaires, Campus Saguenay 305, rue Saint-Vallier, C.P. 221, Chicoutimi, Québec G7H 5H6 Canada

**Keywords:** Value-based healthcare, Health information sytems, Quality improvement, Patient-reported outcomes measures, Organizational decision-making, Review, Learning health system, Patient satisfaction, Patient participation

## Abstract

**Introduction:**

Value-based healthcare (VBHC) implementation is seen as a priority in many healthcare systems worldwide because it advocates for the best health outcomes that matter to patients at the best cost. Patient-reported outcomes measures (PROMs) and patient-reported experience measures (PREMs) are increasingly used to capture this patient’s perspective on their health and their experience of care. Consequently, to promote VBHC, an international emergence of health information systems integrating PROMs and PREMs has developed.

**Objectives:**

The goal of our study was to (1) identify the main existing health information systems integrating PROMs and PREMs and (2) describe how their use supports VBHC and value-based decision-making at meso or macro levels.

**Methods:**

We conducted a scoping review using the Joanna Briggs Institute (JBI) methodology. We conducted a systematic search across MEDLINE, Embase, CINAHL, PsycINFO, Web of Science and Academic Search Complete databases. We carried out a mixed thematic analysis using deductive (frameworks of Franklin et al. and CIHI) and inductive approaches.

**Results:**

We screened 7,497 papers from which the title and abstract of 526 papers were identified as relevant for full-text review. A total of 12 papers were retained for analysis identifying systems from six different countries. We described these systems according to four types of governance strategies. PROMs and PREMs tools were selected according to the population segments chosen. They were collected through paper, phone, and digital technologies. Barriers such as lack of time, administrative burden, reluctance to implementation and facilitators such as identifying champions, integrating patients into the process, efficient communication were described. The real-time reporting of data was an interesting innovation that was developed in several systems. PROMs and PREMs were used for benchmarking, quality improvement, performance evaluation but no concrete example of use were reported.

**Conclusion:**

This scoping review identifies international initiatives of health information systems integrating PROMs and PREMs. The mechanisms use to support value-based decision-making with this data at meso and macro levels are yet to be fully described. Nonetheless, these results will enable countries to learn from the approaches of others to improve the development and implementation of similar initiatives to support value-based healthcare decision-making.

**Supplementary Information:**

The online version contains supplementary material available at https://doi.org/10.1186/s41687-026-01146-6.

## Introduction

There is an international trend towards value-based healthcare (VBHC) [1]. The VBHC model represents an important innovation in healthcare systems to support evidence-based decision making and comprehensiveness in opposition to a volume-based healthcare model [2]. Value in health care is the measured improvement in a patient’s health outcomes for the cost of achieving that improvement [2]. VBHC transformation aims to enable the health care system to create more value for patients [3]. Organizing healthcare around this value improves outcomes that matter to patients while optimizing the use of resources. It also connects clinicians to their purpose as healers, supports their professionalism, and can be a powerful mechanism to counter clinician burnout [3].

The healthcare transformation toward VBHC model implies major changes at micro, meso and macro levels of healthcare institutions (healthcare policies, management of healthcare structures, and the clinician-patient relationship). Therefore, implementing this transformation requires that institutions collect data directly from patients and plan how this data will be collected, stored, processed, visualized and used. Integrating the measurement of health outcomes that matters to patients is one of the first steps in setting up such a data collection [4, 5]. Patient-reported measures of their health outcomes and experience of care, subsequently referred as Patient-Reported Outcomes (PROs) and Patient-Reported Experience (PREs), can inform us about what matters to them and thus guide political and organizational decision-making regarding healthcare system governance [6]. These indicators are captured using scientifically validated measurement tools. Patients are asked to complete these instruments in order to assess, on the one hand, health outcomes (PROMs) and, on the other, patients’ experiences of care across different contexts (PREMs) [6]. These measures are recognized as essential data in improving and evaluating current healthcare systems [7, 8].

Consequently, to promote VBHC, an international emergence of health information system integrating PROMs and PREMs has developed. In the United States, PROMs are part of the mandate of payer organizations (the third party that pays all or part of the medical costs to the physician or reimburses them to the insured) [9]. In the United Kingdom, they are used to benchmark the performance of health and social care services and are included in a national register [10, 11]. In Canada, the Canadian Institute for Health Information (CIHI) has advocated for a standardized pan-Canadian collection of PROMs [12]. The Canadian Partnership Against Cancer (CPAC) recently coordinated the deployment of oncology PROMs in ten provinces/territories [13]. In 2017, the Organisation for Economic Co-operation and Development (OECD) launched the OECD PaRIS international initiative aimed at developing and implementing PROMs and PREMs that are internationally comparable and standardized [14].

Yet the use of these tools remains fragmented, and healthcare systems are at different stages of implementation, ranging from pilot projects to large national initiatives [15, 16]. Implementation is often siloed by local initiatives, leading to duplication and limited support [17]. In addition, there may be differences in how these systems were implemented, for example, whether PROMS or PREMs were an integral part of their development or were introduced later into already existing systems. Also, implementation difficulties linked to barriers at various levels have led to low systemic adoption of PROMs and PREMs in some countries [18, 19].

In this context of transformation towards VBHC, our goal was to describe the main existing health information systems integrating PROMs and PREMs and how their use supports VBHC and value-based decision-making at meso or macro levels.

## Methods

A scoping review was conducted in accordance with the Joanna Briggs Institute methodology [20]. Reporting followed the Preferred Reporting Items for Systematic Reviews and Meta-Analyses for Scoping Review (PRISMA-ScR) checklist [21, 22]. The review protocol was registered on Open Science Framework (https://osf.io/fn6wt).

### Search strategy

We identified studies reporting health information system, integrating PROMs and PREMs, to support value-based decision-making by searching the electronic databases MEDLINE (Ovid), Embase (Elsevier), CINAHL Complete (EBSCO), Academic Search Complete (EBSCO), Web of Science Core Collection (Clarivate), and PsycINFO (EBSCO). The search strategy was developed in collaboration with a librarian (FB) with expertise in literature reviews, and its sensitivity was tested using five articles that the strategy was required to retrieve. Search terms included PROMs, PREMs and quality improvement (Supplementary Table 1). Quality improvement and its synonyms were used as they were commonly employed in the literature to describe initiatives related to value-based decision-making. When available, relevant controlled vocabulary terms were included. The databases were searched from their inception to December 15, 2022.

### Study selection

All studies identified through the search strategy were imported into the systematic review management tool, DistillerSR [23]. Titles and abstracts were independently screened by two reviewers (PHRL, WS), with disagreements resolved by a third reviewer (MS).The screening process was piloted on a sample of 50 articles to assess inter-reviewer agreement. Full-text screening was subsequently conducted independently by three reviewers (PHRL, WS, EA) with disagreements resolved through discussion among all the reviewers (PHRL, WS, EA, MS).

### Study eligibility

Eligible studies were required to: (1) be published in English or French (2), report an initiative involving a health information system integrating PROMs and PREMs and (3) describe the use of PROMs and PREMs to support decision-making at regional, federal or national levels. Non-primary literature, theses, dissertations, conference abstracts, and editorials were excluded.

### Data extraction and synthesis

A data extraction tool was developed using the frameworks proposed by Franklin et al. and the CIHI and implemented in Microsoft Excel [24, 25]. The following data were extracted when available: authors, publication year, journal, study location, design, aims, population characteristics, governance structure, PROMs/PREMs used, data collection methods, end users of PROMs/PREMs for decision-making, type of use, modes of data presentation. Data were synthesized using a mixed thematic analysis combining deductive (based on the Franklin et al. and CIHI frameworks) and inductive approaches [24, 25].

## Results

### Search results

**Fig. 1:**
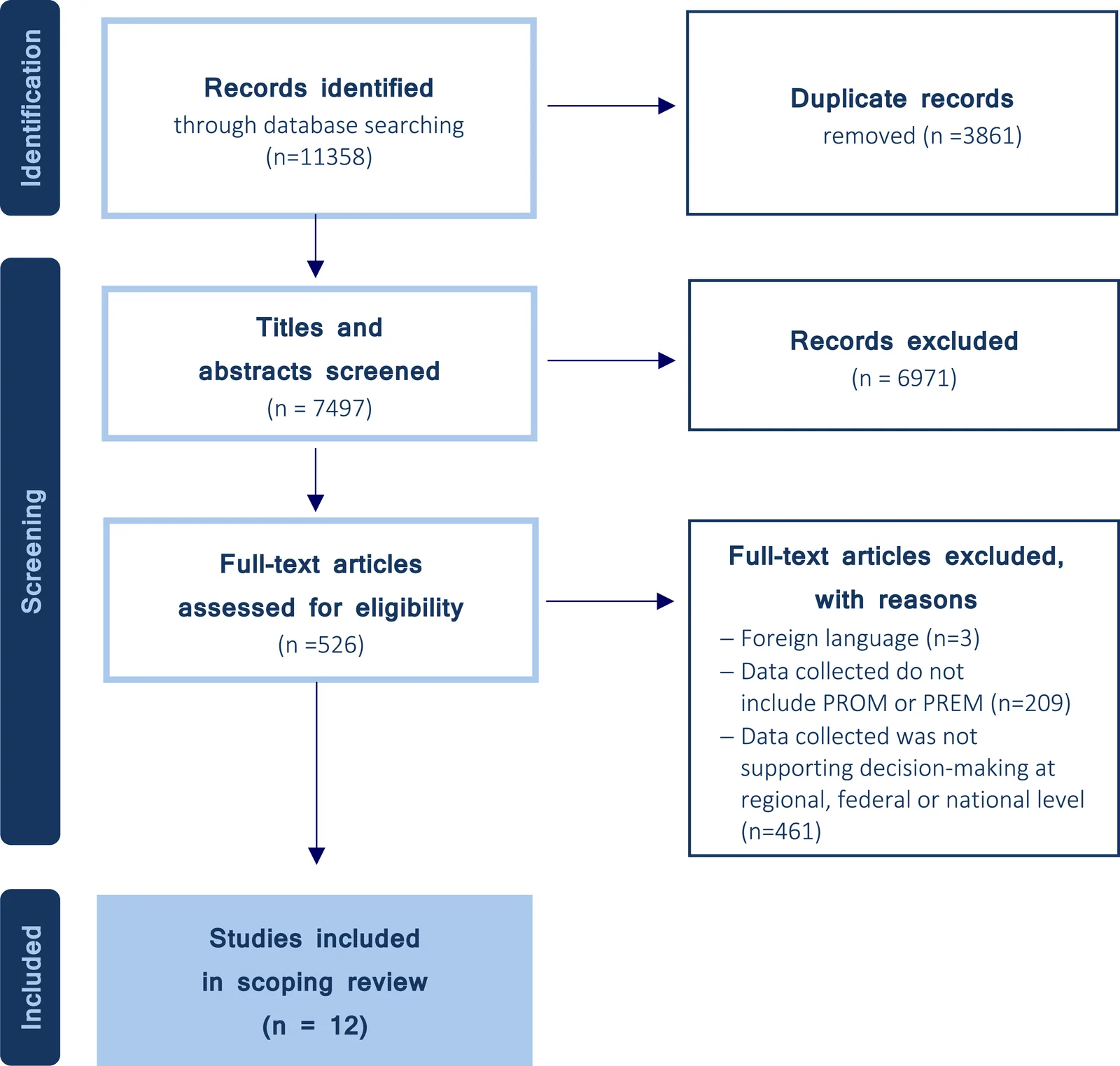
Scoping review flow chart

The search strategy identified 7,497 articles in six databases (Fig. 1). A full-text review was conducted for 526 articles. Twelve articles met the inclusion criteria and were included in this study [26–37].

### Study characteristics

The characteristics of the included studies are shown in Table 1. One study was published in 2010, and the others between 2016 and 2022. Six studies had a quantitative design [26, 28, 32, 33, 35, 37], three a qualitative design [27, 30, 34], one a mixed-method design [36], one used a case study methodology [31] and one a research-action methodology [29]. The initiatives described in the studies were developed in six countries, which are Sweden [27, 28, 31–33, 37], Italy [29, 36], United-Kingdom [30], Denmark [34], Canada [35], Kenya [26]. Half of the studies were conducted in Sweden. Some initiatives were at the pilot stage at the time of the publication [26, 29, 30, 34, 36].

**Table 1:** Study characteristics

Lead author	Country	Study design
Al-Shammari et al., (2019)	Kenya	Quantitative
Devlin et al., (2010)	United Kingdom	Qualitative
Bergerum et al., (2022)	Sweden	Qualitative
Cnudde et al., (2016)	Sweden	Quantitative
Ernestsson et al., (2020)	Sweden	Case study (description of registries)
Feltelius et al., (2017)	Sweden	Quantitative
Glenngård and Anell, (2018)	Sweden	Quantitative
Stranne et al., (2020)	Sweden	Quantitative
Kristensen et al., (2022)	Denmark	Qualitative
De Rosis et al., (2020)	Italy	Action research
Pennucci et al., (2020)	Italy	Mixed-method
Olson et al., (2018)	Canada	Quantitative

### Health information systems characteristics

The characteristics of health information systems integrating PROMs or PREMs are presented in Table 2. Depending on the countries, there were four different governance structures: systems with national governance [30]; systems with an external third party organisation governance in collaboration with the regional government [29, 35, 36]; systems with governance from the national registries [27, 28, 31–34, 37]; and systems with a local team responsible for the governance [26]. There were different types of targeted population. The most frequent were the population with hip and knee replacement [28, 30], cardiologic conditions [31, 32, 36] and cancer conditions [27, 31, 32, 35, 37].

**Table 2:** Health information system characteristics

Lead author	Governance struture	Population	Which PROMs are used?	Which PREMs are used?	How are PROMs/PREMs collected?
Al-Shammari et al., (2019)	Local team	Pregnant women	ICHOM Pregnancy and childbirth standard set adapted for Kenya: ICIQ-SF, Wexner, PROMIS SFFAC102 and PHQ-2.	N/A	Digital platform on phone
Devlin et al., (2010)	National government	Patients receiving surgery (unilateral hip replacement, unilateral knee replacement, groin hernia repair, varicose vein surgery and cataract surgery)	EQ-5D-5 L; EQ-VAS; SF-36; VF-14; Aberdeen Varicose Vein Symptom Severity questionnaire; Oxford Knee Score; Oxford Hip Score	N/A	Paper-based
Bergerum et al., (2022)	National registries	Patient from internal medicine, oncology, paediatric and rehabilitation	N/A	N/A	N/A
Cnudde et al., (2016)	National registries	Hip and knee osteoarthritis	Visual analogue scale (VAS) hip pain; EuroQol 5; EuroQol VAS; Satisfaction VAS	N/A	N/A
Ernestsson et al., (2020)	National registries	Patient with conditions related to the musculoskeletal system, nervous system, circulatory system, psychiatry, cancer, infections, paediatrics, obstetrics and gynaecology, stomach and intestines, endocrine organs, skin diseases, rare diseases	EQ-5D-3 L; EQ-5D-5 L; EQ VAS	N/A	Paper-based, web-based or interview (telephone or face-to-face)
Feltelius et al., (2017)	National registries	Patient with condition related to cardiology, neurology, multiple sclerosis (MS), stroke, rheumatoid arthritis, diabetes, prostate and breast cancer, ophthalmology	N/A	N/A	Web-based, paper-based
Glenngård and Anell, (2018)	National registries	Patients having visited a provider in primary care	N/A	National Patient Survey	Web-based (link sent by e-mail)
Stranne et al., (2020)	National registries	Patient with prostate cancer treated by radical prostatectomy	PROMs not specified: included questions on continence, erectile function and inguinal hernia formation		Web-based, paper-based
Kristensen et al., (2022)	National registries	Patients with anxiety, affective disorders or psychotic disorders	PROMs were designed by a patient peer board to on well-being, social functioning and side effects of medication		Web-based
De Rosis et al., (2020)	External third-party organisation in collaboration with regional government	Patients hospitalized in one of the hospital of Tuscany or Veneto	N/A	PREM including questions on admission process, experience in the ward, discharge phase	Web-based (link sent by e-mail or phone)
Pennucci et al., (2020)	External third-party organisation in collaboration with regional government	Patient with chronic heart failure	The Italian short version of the Kansas City Cardiomyopathy Questionnaire-12 (KCCQ-12); The KCCQ-12 scale; the Italian translation of the Self-Care Heart Failure Index (SCHFI)	a PREM (not precised)	Web-based (link sent by e-mail or phone)
Olson et al., (2018)	External third-party organisation in collaboration with regional government	Patients receiving radiotherapy for bones metastases, for brain metastases, lung cancer, gynecological cancer, head and neck cancer	Questions from FACT BP, FACT-L, EPIC, PRO-CTAE, GI toxicity, EORTC QLQ Cx24, EuroQol EQ-5D-5 L		Tablets or mobile phone with the help of a nurse

### Selecting PROMs and PREMs

Most of the included studies reported health information systems of PROMs [26–28, 30–32, 34–37]. De Rosis et al., Pennucci et al. and Glenngård and Anell presented the collection and use of a PREM [29, 33, 36]. However, in De Rosis et al. and Pennucci et al., the PREM was part of an initiative combining PROMs and PREMs [29, 36].

The PROMs selected were often specific to the trajectory studied. They could be associated with a generic PROM, specifically the EQ-5D-5 L, which was used in four studies [28, 30, 31, 35]. The health information systems described were sometimes connected with other clinical data systems [27, 28, 30–32, 37] or administrative data systems [26–28, 30–32, 37]. PREM questionnaires were already existing national or regional experience surveys [29, 33, 36].

Several strategies were used to select the PROMs and PREMs: a PROM was developed in collaboration with a committee of patient partners, followed by several rounds of validation [34]; an existing PREM questionnaire was enhanced using closed and narrative questions (the questions were worded positively to mitigate negativity bias), enabling the collection of quantitative and qualitative data [29]; tools from the pregnancy and childbirth toolbox produced by the International consortium for health measurement (ICHOM) were adapted to the local Kenyan context [26]. In this instance, the tools selected had been validated with expert resources and stakeholders from both private and public sectors. The choice of the tool also depended on its intended use. Devlin et al. explained that the EQ-5D was chosen for the United-Kingdom PROMs programme because it was widely used for medico-economic evaluations [30]. Additionally, patient preferences and choices had to be considered [34]. Finally, Al-Shammari et al. highlighted the accessibility challenges of acquiring a user licence for specific tools, which are not free access.

### Collecting data from PROMs and PREMs

The data were collected using various channels: web-based [26, 29, 31–37], telephone surveys [31], and paper-based [30]. Only Swedish registries utilized all these means simultaneously [31] as most of the other initiatives employed a web-based strategy, except for the English pilot study (published in 2010). The choice of a web-based strategy was particularly justified in the article by De Rosis et al., as data collection via postal surveys or paper-based are expensive and time-consuming; telephone surveys require brief questionnaires and can produce interviewer bias or selection bias. Therefore, according to them, the web-based strategy appeared to be the optimal option. De Rosis et al. also mentioned that web-based methodology requires brief questionnaires but could be accessible across multiple platforms (web, applications). Additionally, the web-based methodology does not increase selection bias due to increased digital literacy and the use of smartphones and Internet across all populations, including older individuals [29]. Furthermore, data collection could be supported by completion reminders and engagement from clinical teams [26, 29, 36].

### Using PROMs and PREMs

The included studies often described the projected use of different health information system. Still, the description of the implemented utilization of the data for decision-making by national or regional institutions was only briefly detailed (Table 3).

**Table 3:** Use of PROMs and PREMs

Lead author	Who uses PROMs/PREMs for decision-making?	What type of use?	What form does the data take?
Al-Shammari et al., (2019)	Kenyan government	Benchmarking on social and health services performance, quality improvement	Aggredated data and statistics
Devlin et al., (2010)	United-Kingdom Department of Health (DH)	Benchmarking on social and health services performance, quality improvement	Aggredated data, reports and statistics
Bergerum et al., (2022)	National Quality Registers	Benchmarking on social and health services performance, quality improvement	Aggredated data
Cnudde et al., (2016)	National Quality Registers, National board of Health and Welfare, Sweden statistics	Benchmarking on social and health services performance, quality improvement, establishing guidelines	Aggredated data and report
Ernestsson et al., (2020)	National quality registers, governmental agencies, health profesionnals	Benchmarking on social and health services performance, quality improvement, economic evaluation, evaluation of interventions, individual patient consultations	Aggredated data in real-time and annual report
Feltelius et al., (2017)	National quality registers, National board of Health and Welfare	Quality improvement, policy decision-making	Aggredated data in real-time and annual report
Glenngård and Anell, (2018)	Regional government	Benchmarking on social and health services performance, quality improvement	Aggredated data and statistics
Stranne et al., (2020)	National quality registers, governmental agencies, health profesionnals	Benchmarking on social and health services and quality performance, quality improvement	Aggredated in real-time
Kristensen et al., (2022)	National registers	Quality improvement	Aggredated data
De Rosis et al., (2020)	Regional governement	Benchmarking on social and health services and quality improvement	Aggredated data in real-time and annual report
Pennucci et al., (2020)	Regional governement	Benchmarking and measure the performance of health-care providers	Aggredated data in real-time and annual report
Olson et al., (2018)	British Columbia Cancer Agency	Quality improvement	Report

The data from the health information system were mainly used to benchmark the different health services or facilities with each other [26–31, 33, 36, 37], to improve the quality of care [26–37] and for data analysis for research purposes [28, 31]. None of the included studies described a process for using PROMs and PREMs data for value-based decision-making at meso or macro levels. However, PROMs and PREMs were often used at the clinical level to support clinician-patient communication, shared decision-making, monitoring, and patient self-management [29, 31, 35–37]. The results could be used to make small, tangible changes at the local level in the short term, but could lead to improvements in the long term [35]. The use of real-time data enabled quick and accurate changes [29, 31, 36, 37].

### Presenting data to the knowledge users

Data presentation was a key aspect in the various empirical studies since it affects its interpretation, the perception of its usefulness and its utilization. The data in these tools (scores, sub-scores, thresholds) are complex to interpret [30]. The data had to be presented in a clear, intelligible, relevant and informative way for those using it (decision-makers, clinicians, patients) [29, 31, 37]. It was necessary to remember that one way of presenting the data was not necessarily the most accurate for everyone [29, 31].

Four studies reported a preferred presentation in the form of real-time data [29, 31, 32, 37]. This allowed users to receive immediate feedback on results. In particular, it was easier for clinicians to remember the data. This made acting quickly on problems and making lasting changes possible [29]. Clinicians and managers saw the use of real-time data as important and positive.

It was also reported that these data were only relevant to clinicians if they were concerned with the patients in their service or unit. There was no support for clinicians to engage in quality improvement using aggregated data. Data were also integrated into patients’ electronic medical records or other data sources to facilitate their use by clinicians [34]. Annual reports were also produced for decision-makers, and other reports were made available to the public [28–32, 35].

### Facilitators and barriers to implementing the health information system

Various implementation facilitators were described in the included studies. Olson et al. reported that implementing these systems without investing additional resources or increasing the workload of professional staff is possible [35]. The facilitator most often cited in the various initiatives was using champions at different system levels, particularly at the clinician level [34–36]. Having convinced champions promoting the initiative was a major facilitator. A second facilitator was the inclusion of patients in the design and implementation of the health information system [34]. Other facilitators described were: communication with patients and clinicians via different informational material (posters, flyers in the departments) [29, 34], informative document explaining the initiative, its goals and the data protection and privacy policy [29], training and support for clinicians during the implementation phase [34]. Finally, allowing teams flexibility in their approach to data collection, for example, by identifying their own solutions, facilitated appropriation and implementation [35].

Barriers to implementation were also described: concerns and resistance to implementation from the clinical teams, administrative and logistical difficulties, lack of training, time constraints, lack of information technologies, budget limitations, and limited administrative and human resources [29, 34, 35].

### Evaluation of the health information system

The included articles proposed several ways of evaluating the health information system. Al-Shammari et al. described a process using quarterly meetings between clinicians from the participating centres to identify weaknesses and areas for improvement in the system implemented [26]. Kristensen et al. described a participatory qualitative approach involving three groups of informants: patients, clinicians and managers [34]. Finally, De Rosis et al. described the use of two performance indicators for the health information system estimating the participation and the response rate [29].

## Discussion

This scoping review aimed to identify health information systems integrating PROMs and PREMs and describe how it supports value-based decision-making at meso or macro levels. Our findings highlight that the use of PROMs and PREMs for explicit value-based decision-making at meso or macro level remains limited and unevenly developed. Although PROMs and PREMs were described as being used for purposes such as quality improvement and benchmarking, the included studies provided only generic descriptions of these uses, with little detail on how the data were concretely used in practice or what specific decisions or changes they informed.

### Governance of the health information system

Governance was mainly organized either through national registries or through collaborations between governmental authorities and external organizations, reflecting a diversity of institutional arrangements supporting system-level PROMs and PREMs initiatives. This diversity indicates that multiple governance pathways can underpin patient-reported data infrastructures, with governance arrangements being context-dependent rather than based on a single pre-defined model applicable across settings. This interpretation aligns with research indicating that sensitivity to context is a key determinant of successful implementation of PROMs and PREMs infrastructures, as such systems must be adaptable to existing organizational structures, professional practices, and local capacities to enable appropriation and sustained use [38]. Accordingly, registry-led governance, particularly prominent in Scandinavian contexts, may offer a stable organizational framework in settings where such infrastructures are already well established, whereas collaborative governance arrangements may represent contextually appropriate solutions in health systems characterized by greater decentralization [31, 39].

Overall, this diversity of governance models is consistent with the view that effective health system governance requires finding a balance between centralization and decentralization, and may involve movement toward multi-level governance structures that seek to combine system-wide coordination with local autonomy [40].

### Development of the health information system

The predominance of PROMs over PREMs observed in this review is consistent with previous findings [41, 42]. The greater use and standardization of PROMs, compared with PREMs, likely reflects the larger and more established body of research focused on PROM development and validation, which has facilitated their integration into health information systems [43]. Consequently, the limited use of PREMs may restrict the capture of relational and experiential dimensions of care that are central to value-based healthcare. In addition, the included initiatives most often relied on specifics PROMs, frequently complemented by generics PROMs, most commonly the EQ-5D-5 L.

### Use for value-based decision-making

Although PROMs and PREMs were described as being used mainly for benchmarking and quality improvement, none of the included studies described a formalized process through which PROMs and PREMs data directly informed value-based decision-making at meso or macro levels. This findings may be explained by the relatively recent emergence of VBHC and the use of PROMs and PREMs at meso or macro levels, with many initiatives still at early or intermediate stages of implementation rather than fully mature decision-support systems [2, 44–47].

Nevertheless, the use of PROMs and PREMs data at clinical and managerial levels enabled practical, small-scale changes, particularly thanks to real-time data systems that facilitate short- and medium-term improvements [48–51]. Over time, the accumulation of these concrete, incremental changes may contribute to long-term improvements and, thus to system-level value creation [35].

### Implementation of the health information system and best practices

The barriers and facilitators identified in this review align closely with those reported in the broader implementation literature and point toward several best practices for system-level deployment of PROMs and PREMs [18, 52–57]. These include the presence of clinical champions across system levels, early and meaningful patient involvement, flexibility in data collection processes, and sustained training and support for clinicians. Persistent challenges related to time constraints, technological infrastructure, and organizational capacity underscore the need for multimodal implementation strategies tailored to local contexts [58, 59]. In this regard, Olson et al. reported that these systems could be implemented without additional resources or increased workload of when teams were given flexibility to adapt data collection processes to their local context, for example, by identifying their own solutions, thereby facilitating appropriation, implementation and supporting the longer-term sustainability of the innovation [35, 60].

These facilitators and strategies could be selected using the Expert Recommendation Implementing Change (ERIC) framework to support a shared taxonomy and help further implementation [58]. At the system level, the framework developed by Ernst et al. offers structured guidance to assess the maturity of PROMs and PREMs implementation, collection and use, and support system-level planning across different stages of deployment, including cross-country or cross-region comparisons [19].

### Strengths and limitations

To our knowledge, this is the first study aimed at describing the use of health information systems integrating PROMs and PREMs to support value-based decision-making at meso or macro levels. Although, no detailed processes for value-based decision-making were identified, the review provides a structured overview of existing initiatives implemented and their use. This work is important in the context of ongoing health system transformation towards VBHC, with the PROMs and PREMs as the only patient-reported tools available and validated to measure what matters to the people.

However, our study had some limitations. First, the review was restricted to peer-reviewed empirical literature published in English or French, and grey literature was not explored. While a snowballing screening was conducted to mitigate this limitation, relevant initiatives described outside the academic literature (particularly policy-driven or payer-led programs) may have been missed. This may explain the absence of US-based initiatives in this review, despite the well-known use of patient-reported outcomes in reimbursement programs in the United States. Second, some identified initiatives were at a pilot or early implementation stage at the time of publication, which may have limited the availability of recent data on these systems. Third, we had to use synonyms in our research strategy to define the concept of “value-based decision-making” which could result in missing key articles on this topic.

## Conclusion

Patient-reported measures are a core component of a value-based healthcare system. Their integration within a health information system is a maturing innovation. This review offers a portrait of existing initiatives of health information systems integrating patient-reported measures worldwide. The precise mechanisms for utilizing this data to support value-based decision-making at meso and macro levels are yet to be fully described. Nonetheless, our findings provide a robust foundation for the future development and implementation of these systems, thereby advancing VBHC decision-making.

## Supplementary Information

Below is the link to the electronic supplementary material.

Supplementary Material 1

## Data Availability

The datasets used and analyzed during the current study are available from the corresponding author upon reasonable request.

## Abbreviations

IT: Information Technology
PROMs: Patient-reported outcome measures
PREMs: Patient-reported experience measures
VBHC: Value-Based HealthCare
