# Supplementary material for: Health information systems integrating patient-reported outcomes and experience: a scoping review
Source: J Patient Rep Outcomes. 2026 Jul 18;10:189. doi: 10.1186/s41687-026-01146-6 (PMC13672266; doi:10.1186/s41687-026-01146-6)
Supplement: Supplementary file 1 [file 41687_2026_1146_MOESM1_ESM.docx]

**Supplementary materials – Database search strategy**

**Medline (OVID)**

**Date of the search:** 15-12-2022

**Database limit:** no database limit has been applied

| **#** | **Search strategy** | **Results** |
| --- | --- | --- |
| 1 | Patient Reported Outcome Measures/ | 12635 |
| 2 | ((patient? OR self) adj2 report* adj2 (outcome? OR Experience?) adj2 measur*).ti,ab,kw,kf OR PREM?.ti,ab,kw,kf OR PROM?.ti,ab,kw,kf | 20531 |
| 3 | 1 OR 2 | 28082 |
| 4 | Quality of Health Care/ OR Quality Improvement/ OR Quality Indicators, Health Care/ OR Quality Control/ OR Quality Assurance, Health Care/ OR Total Quality Management/ OR Registries/ OR Critical Pathways/ | 339254 |
| 5 | (Quality adj2 (Improv* OR Healthcare OR Care OR Indicator?)).ti,ab,kw,kf OR (performance adj4 (assess* OR measur* OR evaluat*)).ti,ab,kw,kf OR ((improv* OR optimiz*) adj2 pathway?).ti,ab,kw,kf OR "Quality Regist*".ti,ab,kw,kf | 397932 |
| 6 | 4 OR 5 | 670661 |
| 7 | 3 AND 6 | 3103 |

**Embase (Embase.com)**

**Date of the search:** 15-12-2022

**Database limit:** results were limited to the Embase database and to prepublications

| **#** | **Search strategy** | **Results** |
| --- | --- | --- |
| 1 | 'patient-reported outcome'/de | 45,598 |
| 2 | ((patient$ OR self) NEAR/2 report* NEAR/2 (outcome$ OR experience$) NEAR/2 measur*):ti,ab,kw OR PREM$:ti,ab,kw OR PROM$:ti,ab,kw | 30,013 |
| 3 | #1 OR #2 | 61,944 |
| 4 | 'health care quality'/de OR 'performance measurement system'/de OR 'quality control'/de OR 'total quality management'/de OR 'register'/exp OR 'clinical pathway'/de | 733,936 |
| 5 | (Quality NEAR/2 (Improv* OR Healthcare OR Care OR Indicator?)):ti,ab,kw OR (performance NEAR/4 (assess* OR measur* OR evaluat*)):ti,ab,kw OR ((improv* OR optimiz*) NEAR/2 pathway?):ti,ab,kw OR "Quality Regist*":ti,ab,kw | 544,699 |
| 6 | #4 OR #5 | 1,148,555 |
| 7 | #3 AND #6 | 7,154 |
| 8 | #7 AND [embase]/lim NOT ([embase]/lim AND [medline]/lim) | 3,089 |

**CINAHL**

**Date of the search:** 15-12-2022

**Database limit:** no database limit has been applied

| **#** | **Search strategy** | **Results** |
| --- | --- | --- |
| 1 | MH "Patient-Reported Outcomes" | 5,175 |
| 2 | TI ((patient# OR self) N2 report* N2 (outcome# OR experience#) N2 measur*) OR AB ((patient# OR self) N2 report* N2 (outcome# OR experience#) N2 measur*) OR TI PREM# OR AB PREM# OR TI PROM# OR AB PROM# | 9,736 |
| 3 | S1 OR S2 | 13,391 |
| 4 | MH "Quality of Health Care" OR MH "Quality Improvement" OR MH"Quality Management, Organizational" OR MH "Critical Path" | 144,583 |
| 5 | TI (Quality N2 (Improv* OR Healthcare OR Care OR Indicator#)) OR AB (Quality N2 (Improv* OR Healthcare OR Care OR Indicator#)) OR TI (performance N4 (assess* OR measur* OR evaluat*)) OR AB (performance N4 (assess* OR measur* OR evaluat*)) OR TI ((improv* OR optimiz*) N2 pathway#) OR AB ((improv* OR optimiz*) N2 pathway#) OR TI "Quality Regist*" OR AB "Quality Regist*" | 186,050 |
| 6 | S4 OR S5 | 286,921 |
| 7 | S3 AND S6 | 1,452 |

**Academic Search Premier**

**Date of the search:** 15-12-2022

**Database limit:** no database limit has been applied

| **#** | **Search strategy** | **Results** |
| --- | --- | --- |
| 1 | DE "PATIENT reported outcome measures" | 1,578 |
| 2 | TI ((patient# OR self) N2 report* N2 (outcome# OR experience#) N2 measur*) OR AB ((patient# OR self) N2 report* N2 (outcome# OR experience#) N2 measur*) OR KW ((patient# OR self) N2 report* N2 (outcome# OR experience#) N2 measur*) OR TI PREM# OR AB PREM# OR KW PREM# OR TI PROM# OR AB PROM# OR KW PROM# | 21,086 |
| 3 | S1 OR S2 | 21,605 |
| 4 | DE "MEDICAL quality control" OR DE "TOTAL quality management" OR DE "QUALITY control" OR DE "MEDICAL quality control" OR DE "TOTAL quality control" | 58,718 |
| 5 | TI (Quality N2 (Improv* OR Healthcare OR Care OR Indicator#)) OR AB (Quality N2 (Improv* OR Healthcare OR Care OR Indicator#)) OR KW (Quality N2 (Improv* OR Healthcare OR Care OR Indicator#)) OR TI (performance N4 (assess* OR measur* OR evaluat*)) OR AB (performance N4 (assess* OR measur* OR evaluat*)) OR KW (performance N4 (assess* OR measur* OR evaluat*)) OR TI ((improv* OR optimiz*) N2 pathway#) OR AB ((improv* OR optimiz*) N2 pathway#) OR KW ((improv* OR optimiz*) N2 pathway#) OR TI "Quality Regist*" OR AB "Quality Regist*" OR KW "Quality Regist*" | 493,333 |
| 6 | S4 OR S5 | 532,549 |
| 7 | S3 AND S6 | 1,329 |

**PsycInfo (OVID)**

**Date of the search:** 15-12-2022

**Database limit:** no database limit has been applied

| **#** | **Search strategy** | **Results** |
| --- | --- | --- |
| 1 | Patient Reported Outcome Measures/ | 626 |
| 2 | ((patient? OR self) adj2 report* adj2 (outcome? OR Experience?) adj2 measur*).ti,ab,id OR PREM?.ti,ab,id OR PROM?.ti,ab,id | 2922 |
| 3 | 1 OR 2 | 3031 |
| 4 | Quality of Health Care/ OR Quality Improvement/ OR Quality Indicators, Health Care/ OR Quality Control/ OR quality assurance, health care/ OR total quality management/ OR Registries/ OR Critical Pathways/ | 2138 |
| 5 | (Quality adj2 (Improv* OR Healthcare OR Care OR Indicator?)).ti,ab,id OR (performance adj4 (assess* OR measur* OR evaluat*)).ti,ab,id OR ((improv* OR optimiz*) adj2 pathway?).ti,ab,id OR "Quality Regist*".ti,ab,id | 98873 |
| 6 | 4 OR 5 | 100178 |
| 7 | 3 AND 6 | 276 |

**Web of Science**

**Date of the search:** 15-12-2022

**Database limit:** no database limit has been applied

| **#** | **Search strategy** | **Results** |
| --- | --- | --- |
| 1 | TS=((patient$ OR self) NEAR/2 report* NEAR/2 (outcome$ OR experience$) NEAR/2 measur*) OR TS=PREM$ OR TS=PROM$ | 25,697 |
| 2 | TS=(Quality NEAR/2 (Improv* OR Healthcare OR Care OR Indicator?)) OR TS=(performance NEAR/4 (assess* OR measur* OR evaluat*)) OR TS=((improv* OR optimiz*) NEAR/2 pathway?) OR TS="Quality Regist*" | 1,114,494 |
| 3 | #1 AND #2 | 2,109 |
